# Steady State Response Analysis of a Tubular Piezoelectric Print Head

**DOI:** 10.3390/s16010081

**Published:** 2016-01-12

**Authors:** Jiaqing Chang, Yaxin Liu, Bo Huang

**Affiliations:** State Key Laboratory of Robotics and System, Harbin Institute of Technology, Harbin 150001, China; cjq2018@outlook.com (J.C.); liuyaxin@hit.edu.cn (Y.L.)

**Keywords:** tubular piezoelectric print head, piezoelectric tube, glass tube, wall thickness

## Abstract

In recent years, inkjet technology has played an important role in industrial materials printing and various sensors fabrication, but the mechanisms of the inkjet print head should be researched more elaborately. The steady state deformation analysis of a tubular piezoelectric print head, which can be classified as a plane strain problem because the radii of the tubes are considerably smaller than the lengths, is discussed in this paper. The geometric structure and the boundary conditions are all axisymmetric, so a one-dimensional mathematical model is constructed. By solving the model, the deformation field and stress field, as well as the electric potential distribution of the piezoelectric tube and glass tube, are obtained. The results show that the deformations are on the nanometer scale, the hoop stress is larger than the radial stress on the whole, and the potential is not linearly distributed along the radial direction. An experiment is designed to validate these computations. A discussion of the effect of the tubes’ thicknesses on the system deformation status is provided.

## 1. Introduction

Ink jet technology has been widely used in the fabrication of integrated circuits (ICs) [1], polymer light emitting displays [2], sample preparation [3], microelectromechanical systems (MEMS) [4], cell printing [5,6], and the fabrication of different sensors [7,8,9]. Numerous excellent published papers have accounted for various applications of ink jet technology, but few of these papers are related to the action mechanism. To fully understand this physical phenomenon, and to obtain some useful instructions in print head design, it is very necessary to determine the mechanism for ink jet technology. This paper studies the action mechanism of a tubular piezoelectric print head, which is typically represented by the mechanical device designed by Zoltan [10].

Dijksman [11] firstly constructed an important model for the dynamic analysis of the tubular print head. Starting at the vibration amplitude and frequency of the inner glass tube, he successfully obtained the speed of the meniscus at the nozzle outlet in the time and frequency domain. The tubular print head was divided into nine segments of different radii or constraints, and the motion of the inner glass tube was decomposed into 250 terms of sinusoidal vibration through Fourier decomposition. Shin [12] analyzed the velocity and stress distribution. The analytical axial velocity history was used as the initial condition when doing his numerical simulation of the jetting and droplet formation process. Fabrication effects, such as the eccentricity alignment and the configuration of the electrode layer on the calculation results based on the model proposed, was also discussed. However, the stress and deformation distributions in piezoelectric tube and glass tube, as well as the influence of the relative thickness of these two tubes on the deformation status, were not provided in their work. Wijshoff [13] presented the actuator design of a piezoelectric print head and conducted the deformation analysis of the channels bonded to the piezoelectric element. However, the analysis was mainly about the piezoelectric print heads of bump or bend modes.

Kim [14] analyzed the pressure waves in a silicon MEMS-fabricated printhead under the excitation of a trapezoid voltage waveform. Numerical simulations and lumped element modeling were conducted, and results show that the Helmholtz mode is the dominant resonance mode acting on the flow oscillations at the nozzle. Bugdayci [15] discussed the quasi-static motion of a piezoelectric tube filled with liquid but did not consider the effect of the glass tube. Moreover, when considering the resulting fluid pressure in the glass tube, it was assumed that both ends of the glass tube were completely sealed. However, the actual situation was that one end was connected to a plastic hose, and the other end was connected to an aperture plate, which made the pressure caused by fluid compression considerably smaller. Larbi [16] developed a model that accounted for for the free vibration of a simply-supported arbitrarily thick laminated piezoceramic cylinder completely filled with fluid. The piezoelectric layers of the laminated cylinder are polarized in the radial direction. Chen [17] investigated the dynamic process of a hollow cylinder filled with compressible fluid based on three-dimensional state space formulations. The free vibration of a multi-layered piezoelectric hollow cylinder and the wave propagation in an infinite homogeneous cylinder were therefore obtained.

Under the excitation of a step voltage, the piezoelectric tube causes the coupling glass tube to deform and squeezes the internal fluid out of the nozzle aperture. Aimed at the above process, this paper analyzes the deformation and stress statuses of piezoelectric and glass tubes after the input of electric voltage. The obtained results could help us understand the action mechanism of the liquid jet process, determine the deformation and stress statuses when an electric voltage is input, and guide us to design a proper piezoelectric print head.

**Figure 1:**
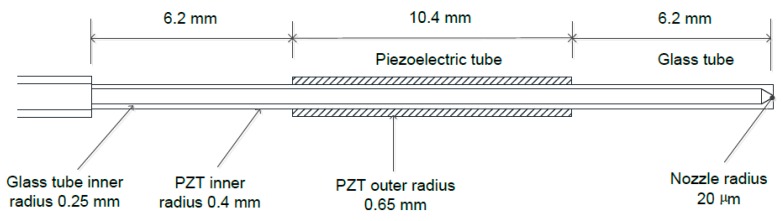
Configuration of the print head.

A tubular piezoelectric print head, as shown in Figure 1, is used to validate these calculations. The mechanical size parameters are also shown in Figure 1. This kind of single nozzle piezoelectric print head is made and sold by the Microdrop and MicroFab companies. About the model, some simplifications and uncertainties are stated as follows. The epoxy layer is omitted in this model because this layer often measures 10–25 μm in thickness (refer to [18] for details about the fabrication process) and is relatively thin. Furthermore, in steady state analysis, this layer only transmits forces, so it does not have much effect on the ultimate deformation and stress statuses. Another aspect needing consideration is that part of the piezoelectric tube is wrapped by a thin electrode layer, and the wrapped segment of the piezoelectric tube (about 2 mm in length) will not generate an electric field and will not have a driving effect. The shear deformation and shear stress are also neglected in the constructed model.

## 2. Mathematical Model Construction

**Figure 2:**
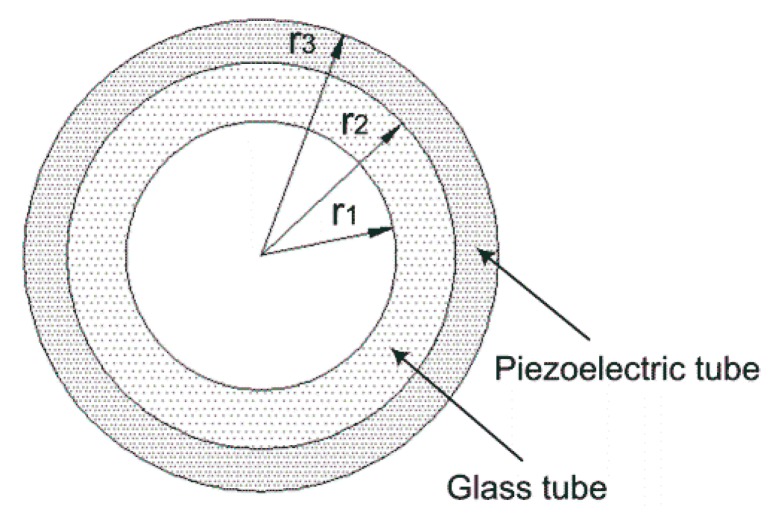
The cross section of the tubular piezoelectric print head.

The cross section of the tubular piezoelectric print head is shown in Figure 2. Owing to its geometric shape, a cylindrical coordinate system is adopted. The deformation and stress statuses of a radially polarized piezoelectric ceramic tube under the excitation of step voltage are studied in this paper. When performing the steady state deformation and stress analysis, the axisymmetric conditions enable $u_{\theta }=0$, $u_{z}=u_{z}(r,z,t)$, $u_{r}=u_{r}(r,z,t)$, and φ=φ(r,z,t). Moreover, because the length of the piezoelectric tube is relatively larger than the radius, it is suitable to classify it as a plane strain problem, so that $u_{\theta }=u_{z}=0$, $u_{r}=u_{r}(r,t)$ and φ=φ(r,t) are obtained. Then, the strain relationship is simplified as

(1) $$\gamma_{rr}=\partial u_{r}/\partial r,\gamma_{\theta \theta }=u_{r}/r$$

Piezoelectric constitutive equations become

(2) $$\sigma_{rr}=c_{33}u_{r,r}+c_{13}\frac{u_{r}}{r}+e_{33}\varphi_{,r}$$

(3) $$\sigma_{\theta \theta }=c_{13}u_{r,r}+c_{11}\frac{u_{r}}{r}+e_{31}\varphi_{,r}$$

(4) $$D_{r}=e_{33}u_{r,r}+e_{31}\frac{u_{r}}{r}-\varepsilon_{33}\varphi_{,r}$$

The stress equation is shown as

(5) $$\sigma_{rr,r}+\frac{1}{r}\left(\sigma_{rr}-\sigma_{\theta \theta }\right)=\rho \frac{\partial^{2}u_{r}}{\partial t^{2}}$$

The steady state response is only studied in this paper, so the inertial term is discarded.

(6) $$\sigma_{rr,r}+\frac{1}{r}\left(\sigma_{rr}-\sigma_{\theta \theta }\right)=0$$

Since there is no free charge inside the piezoelectric tube, the Gauss equation becomes

(7) $$\frac{1}{r}\frac{\partial }{\partial r}(rD_{r})=0$$

From Equation (7), it can be inferred that

(8) Dr=A/r,(A is a constant)

From Equation (4), the following equations are deduced

(9) $$\varphi_{,r}=\frac{e_{33}}{\varepsilon_{33}}u_{r,r}+\frac{e_{31}}{\varepsilon_{33}}\frac{u_{r}}{r}-\frac{A}{\varepsilon_{33}r}$$

(10) $$\varepsilon_{33}\varphi =e_{33}u_{r}+e_{31}\int \frac{u_{r}}{r}dr-A\ln r+k_{1}$$

where $k_{1}$ is an undetermined constant.

By substituting Equations (2) and (3) into Equation (6),

(11) $$c_{33}\left(u_{r,rr}+\frac{1}{r}u_{r,r}\right)-c_{11}\frac{1}{r^{2}}u_{r}+e_{33}\varphi_{,rr}+\left(e_{33}-e_{31}\right)\frac{1}{r}\varphi_{,r}=0$$

After substituting Equation (9) into Equation (11),

(12) $$u_{r,rr}+\frac{1}{r}u_{r,r}-\frac{\nu^{2}}{r^{2}}u_{r}=\frac{k_{4}}{r^{2}}$$

where $\nu^{2}={\bar{c}}_{11}/{\bar{c}}_{33}$; $k_{4}=-\left(e_{31}/{\bar{c}}_{33}\varepsilon_{33}\right)A$; ${\bar{c}}_{11}=c_{11}+\left(e_{31}^{2}/\varepsilon_{33}\right)$; ${\bar{c}}_{33}=c_{33}+\left(e_{33}^{2}/\varepsilon_{33}\right)$.

The solution of Equation (12) is

(13) $$u_{r}=k_{2}r^{\nu }+k_{3}r^{-\nu }-\frac{k_{4}}{\nu^{2}}$$

It is assumed that the electrical potential of the inner electrode for the piezoelectric tube is zero, and the electrical potential of the outer electrode is $V_{in}$. The thickness of the electrodes is neglected. Substituting Equation (13) into Equation (10), the electrical potential of the inner electrode is given in Equation (14).

(14) $$k_{1}+e_{33}{r_{2}}^{\nu }k_{2}+e_{33}{r_{2}}^{-\nu }k_{3}+\left(\frac{{\bar{c}}_{33}\varepsilon_{33}}{e_{31}}\ln r_{2}-\frac{e_{33}}{\nu^{2}}\right)k_{4}=0$$

In addition, the electrical potential at $r=r_{3}$ is given by

(15) $$e_{33}\left(k_{2}{r_{3}}^{\nu }+k_{3}{r_{3}}^{-\nu }-\frac{k_{4}}{\nu^{2}}\right)+e_{31}\left(k_{2}\frac{r_{3}^{\nu }}{\nu }-k_{3}\frac{r_{3}^{-\nu }}{\nu }-\frac{k_{4}}{\nu^{2}}\ln r_{3}-k_{2}\frac{r_{2}^{\nu }}{\nu }+k_{3}\frac{r_{2}^{-\nu }}{\nu }+\frac{k_{4}}{\nu^{2}}\ln r_{2}\right)-A\ln r_{3}+k_{1}=\varepsilon_{33}V_{in}$$

From Equations (2) and (9), Equation (16) is derived

(16) $$\sigma_{rr}={\bar{c}}_{33}u_{r,r}+{\bar{c}}_{13}\frac{u_{r}}{r}-\frac{e_{33}A}{\varepsilon_{33}r}$$

where ${\bar{c}}_{13}=c_{13}+\frac{e_{31}e_{33}}{\varepsilon_{33}}$.

The free boundary condition of the outer surface of the piezoelectric tube makes the stress become zero.

(17) $$\sigma_{rr}(r_{3})=\left({\bar{c}}_{33}\nu r_{3}^{\nu -1}+{\bar{c}}_{13}r_{3}^{\nu -1}\right)\cdot k_{2}+\left[{\bar{c}}_{13}r_{3}^{-(\nu +1)}-{\bar{c}}_{33}\nu r_{3}^{-(\nu +1)}\right]\cdot k_{3}+\left(\frac{{\bar{c}}_{33}e_{33}}{e_{31}r_{3}}-\frac{{\bar{c}}_{13}}{\nu^{2}r_{3}}\right)\cdot k_{4}=0$$

The stress on the inner surface of the piezoelectric tube, represented as $P_{2}$, is outward because of the deformation resistance from the glass tube. Then,

(18) $$\sigma_{rr}(r_{2})=\left({\bar{c}}_{33}\nu r_{2}^{\nu -1}+{\bar{c}}_{13}r_{2}^{\nu -1}\right)\cdot k_{2}+\left[{\bar{c}}_{13}r_{2}^{-(\nu +1)}-{\bar{c}}_{33}\nu r_{2}^{-(\nu +1)}\right]\cdot k_{3}+\left(\frac{{\bar{c}}_{33}e_{33}}{e_{31}r_{2}}-\frac{{\bar{c}}_{13}}{\nu^{2}r_{2}}\right)\cdot k_{4}=P_{1}$$

**Figure 3:**
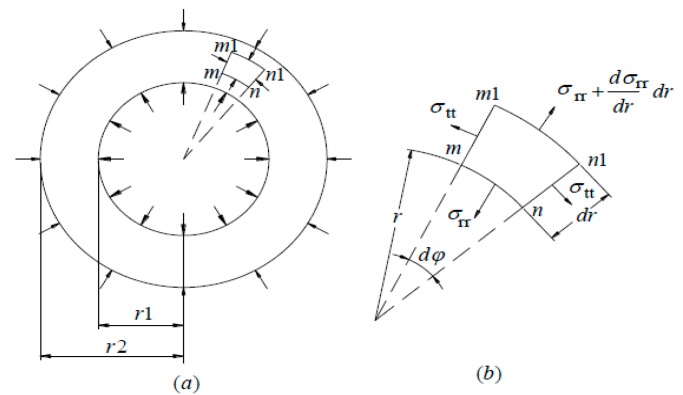
Force diagram of the glass tube.

To simplify the analysis, the glass tube is viewed as an isotropic material. This is suitable in deformation analysis. Timoshenko described the steady state force equation of an isotropic thick-wall hollow cylinder in Reference [19], and the force diagram is depicted in Figure 3. The following analysis quotes the equations in Reference [19] directly.

The radial displacement function of the glass tube is

(19) $$u_{r}^{\prime }=k_{5}r+\frac{k_{6}}{r}$$

The stress on the outer surface of the glass tube is negative for it behaves similar to a compressive stress and equals the normal stress on the inner surface of the piezoelectric tube in magnitude. Therefore

(20) $$\sigma_{rr}^{\prime }(r_{2})=\frac{E}{1-\mu^{2}}\left[k_{5}(1+\mu )-k_{6}\frac{1-\mu }{r_{2}^{2}}\right]=-P_{1}$$

From Equations (18) and (20), it can be deduced that

(21) $$\begin{array}{l} \left({\bar{c}}_{33}\nu r_{2}^{\nu -1}+{\bar{c}}_{13}r_{2}^{\nu -1}\right)\cdot k_{2}+\left[{\bar{c}}_{13}r_{2}^{-(\nu +1)}-{\bar{c}}_{33}\nu r_{2}^{-(\nu +1)}\right]\cdot k_{3}\\ +\left(\frac{{\bar{c}}_{33}e_{33}}{e_{31}r_{2}}-\frac{{\bar{c}}_{13}}{\nu^{2}r_{2}}\right)\cdot k_{4}+\frac{E}{1-\mu^{2}}\left[k_{5}(1+\mu )-k_{6}\frac{1-\mu }{r_{2}^{2}}\right]=0 \end{array}$$

As for the inner surface of the glass tube, although fluid inside it would resist the deformation of the glass tube, the fluid pressure would eventually go to zero because the ends of the glass tube are not sealed. Therefore, the radial stress on the inner surface of the glass tube is zero.

(22) $$\frac{E}{1-\mu^{2}}\left[k_{5}(1+\mu )-k_{6}\frac{1-\mu }{r_{1}^{2}}\right]=0$$

The hoop stress of the glass tube is shown as

(23) $$\sigma_{\theta \theta }^{\prime }(r)=\frac{E}{1-\mu^{2}}\left[k_{5}(1+\mu )+k_{6}\frac{1-\mu }{r_{2}^{2}}\right]$$

The displacement of the outer surface of the glass tube is

(24) $$u_{r2}^{\prime }=k_{5}r_{2}+\frac{k_{6}}{r_{2}}$$

Considering the bonding condition between the outer surface of the glass tube and the inner surface of the piezoelectric tube, Equation (25) is consequently obtained.

(25) $$k_{2}{r_{2}}^{\nu }+k_{3}{r_{2}}^{-\nu }-\frac{k_{4}}{\nu^{2}}-\left(k_{5}r_{2}+\frac{k_{6}}{r_{2}}\right)=0$$

## 3. Solving Method and Results

Next, by combining Equations (14), (15), (17), (21), (22) and (25), the following equation is obtained

(26) H⋅K=Y

where

$$H=\left[\begin{array}{cccccc} 1 & e_{33}r_{2}^{\nu } & e_{33}r_{2}^{-\nu } & \frac{{\bar{c}}_{33}\varepsilon_{33}}{e_{31}}\ln r_{2}-\frac{e_{33}}{\nu^{2}} & 0 & 0\\ 1 & e_{33}r_{3}^{\nu }+\frac{e_{31}}{\nu }(r_{3}^{\nu }-r_{2}^{\nu }) & e_{33}r_{3}^{-\nu }+\frac{e_{31}}{\nu }(r_{2}^{-\nu }-r_{3}^{-\nu }) & \frac{1}{\nu^{2}}\left(e_{31}\ln r_{2}-e_{31}\ln r_{3}-e_{33}\right)+\frac{{\bar{c}}_{33}\varepsilon_{33}}{e_{31}}\ln r_{3} & 0 & 0\\ 0 & \left({\bar{c}}_{33}\nu +{\bar{c}}_{13}\right)r_{3}^{\nu -1} & \left({\bar{c}}_{13}-{\bar{c}}_{33}\nu \right)r_{3}^{-(\nu +1)} & \left(\frac{{\bar{c}}_{33}e_{33}}{e_{31}}-\frac{{\bar{c}}_{13}}{\nu^{2}}\right)\frac{1}{r_{3}} & 0 & 0\\ 0 & \left({\bar{c}}_{33}\nu +{\bar{c}}_{13}\right)r_{2}^{\nu -1} & \left({\bar{c}}_{13}-{\bar{c}}_{33}\nu \right)r_{2}^{-(\nu +1)} & \left(\frac{{\bar{c}}_{33}e_{33}}{e_{31}}-\frac{{\bar{c}}_{13}}{\nu^{2}}\right)\frac{1}{r_{2}} & \frac{E}{1-\mu } & -\frac{E}{(1+\mu )r_{2}^{2}}\\ 0 & r_{2}^{\nu } & r_{2}^{-\nu } & -\frac{1}{\nu^{2}} & -r_{2} & -\frac{1}{r_{2}}\\ 0 & 0 & 0 & 0 & \frac{E}{1-\mu } & -\frac{E}{(1+\mu )r_{1}^{2}} \end{array}\right]$$

$$K={\left[\begin{array}{cccccc} k_{1} & k_{2} & k_{3} & k_{4} & k_{5} & k_{6} \end{array}\right]}^{T},\;Y={\left[\begin{array}{cccccc} 0 & \varepsilon_{33}V_{in} & 0 & 0 & 0 & 0 \end{array}\right]}^{T}$$

The material parameters for piezoelectric material PZT-5H are shown in the appendix of Reference [20]. The elastic modulus and Poisson’s ratio of the Pyrex heat-resistant glass tube is 61.67 GPa (see Reference [21]) and 0.2, respectively. Additionally, the radii of the tubes are: r1=2.5×10−4 m, r2=4.0×10−4 m, and r3=6.5×10−4 m. Simultaneous Equation (26) is calculated and rewritten as

(27) $$\left[\begin{array}{cccccc} 1.0 & 1.9396\times 10^{-2} & 2.7846\times 10^{4} & 2.559\times 10^{3} & 0 & 0\\ 1.0 & 2.7\times 10^{-2} & 1.5005\times 10^{4} & 2.404\times 10^{3} & 0 & 0\\ 0 & 4.344\times 10^{11} & -8.31\times 10^{16} & -1.048\times 10^{15} & 0 & 0\\ 0 & 4.534\times 10^{11} & -2.096\times 10^{17} & -1.703\times 10^{15} & 7.71\times 10^{10} & -3.212\times 10^{17}\\ 0 & 8.346\times 10^{-4} & 1.1982\times 10^{3} & -1.22 & -4.0\times 10^{-4} & -2.5\times 10^{3}\\ 0 & 0 & 0 & 0 & 7.71\times 10^{10} & -8.224\times 10^{17} \end{array}\right]\left[\begin{array}{c} k_{1}\\ k_{2}\\ k_{3}\\ k_{4}\\ k_{5}\\ k_{6} \end{array}\right]=\left[\begin{array}{c} 0\\ 1.3\times 10^{-8}V_{in}\\ 0\\ 0\\ 0\\ 0 \end{array}\right]$$

The solution is not correct if the above equation is typed into the calculation software directly because of the effective numeral problem. Therefore, it is essential to do the following transformation.

(28) $$x_{1}=10^{8}k_{1},\;x_{2}=10^{6}k_{2},\;x_{3}=10^{12}k_{3},\;x_{4}=10^{10}k_{4},\;x_{5}=10^{5}k_{5},\;x_{6}=10^{12}k_{6}$$

Suppose the input voltage is 1.0 V, *i.e.*, $V_{in}=1.0$. Eventually, the following equation can be obtained such that

(29) $$\begin{array}{c} k_{1}=-1.643\times 10^{-7},\;k_{2}=-2.079\times 10^{-7},\;k_{3}=-2.222\times 10^{-12},\;\\ k_{4}=8.997\times 10^{-11},\;k_{5}=-4.642\times 10^{-6},\;k_{6}=-4.352\times 10^{-13} \end{array}$$

**Figure 4:**
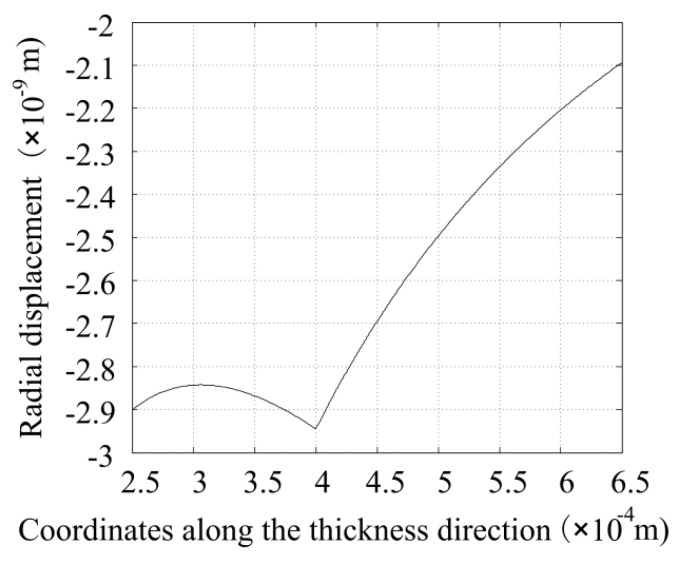
Displacement distribution along the thickness direction when the voltage amplitude is 1 V.

Substituting these obtained coefficients into the aforementioned equations, some meaningful results occur. Utilizing Equations (13) and (19), the displacement function along the thickness direction is obtained, as plotted in Figure 4. It can be found that the displacement of the inner surface of the piezoelectric tube equals the displacement at the outer surface of the glass tube, which shows the correctness of the results. The values are all negative, which means that the tubes are all displaced toward the central axis when the input voltage is positive. As shown in Figure 4, the outer surface of the piezoelectric tube has a minimum displacement of 2.1 nm, and the displacement at the location where the piezoelectric and glass tubes adhere together has a maximum displacement of 2.94 nm. These displacement results seem larger than the usually known values. We cautiously believe that this is because the step voltage signal is different from a train of trapezoid pulses from the perspective of the Rayleigh’s energy.

**Figure 5:**
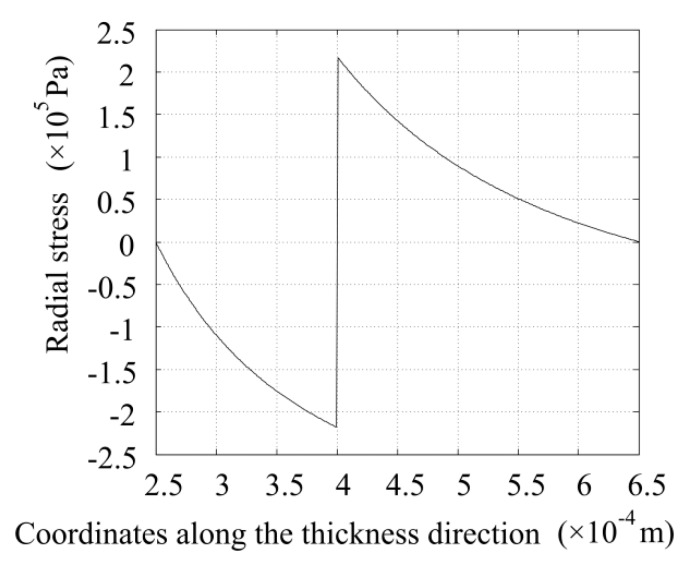
Radial stress distribution along the thickness direction when the voltage amplitude is 1 V.

Exploiting Equations (18) and (20), the radial stress distribution along the thickness direction is obtained and is shown in Figure 5. The radial stresses in the glass and piezoelectric tubes are negative and positive, respectively, which means that the radial stresses in the glass tube point inward. For the piezoelectric tube, the radial stresses point outward. The radial stresses at the inner surface of the glass tube and the outer surface of the piezoelectric tube are all zero. The stress at the outer surface of the glass tube equals the stress at the inner surface of the piezoelectric tube, but with an opposite direction. These results all conform to the known conditions and show the correctness of the obtained results.

**Figure 6:**
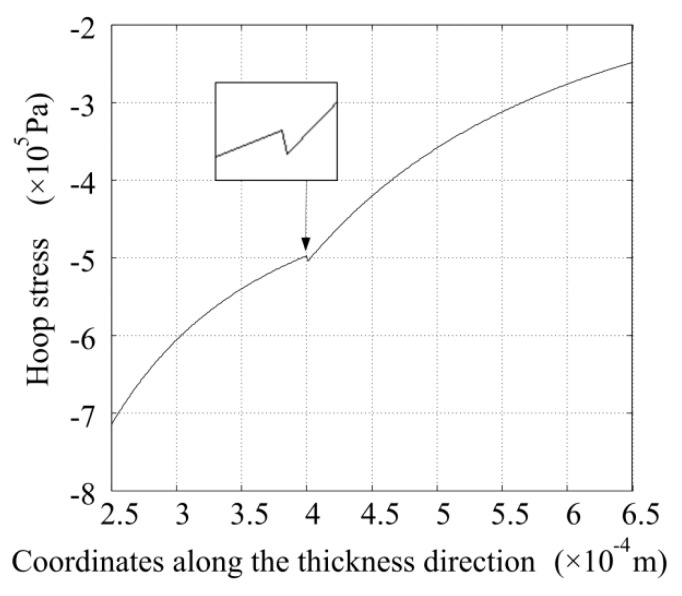
Hoop stress distribution along the thickness direction when the voltage amplitude is 1 V.

Utilizing Equations (3) and (23), the hoop stress distribution along the thickness direction is obtained and is shown in Figure 6. The hoop stresses in the glass and piezoelectric tubes are all negative. This means that the particles in the tubes in the circumferential direction are all compressed. The hoop stress increases as the radius decreases and reaches the maximum at the inner surface of the glass tube. The hoop stress is not continuous at the place where the two tubes adhere together, which conforms to physical truth. In Figure 6 it can be found that the hoop stress is generally larger than the radial stress.

**Figure 7:**
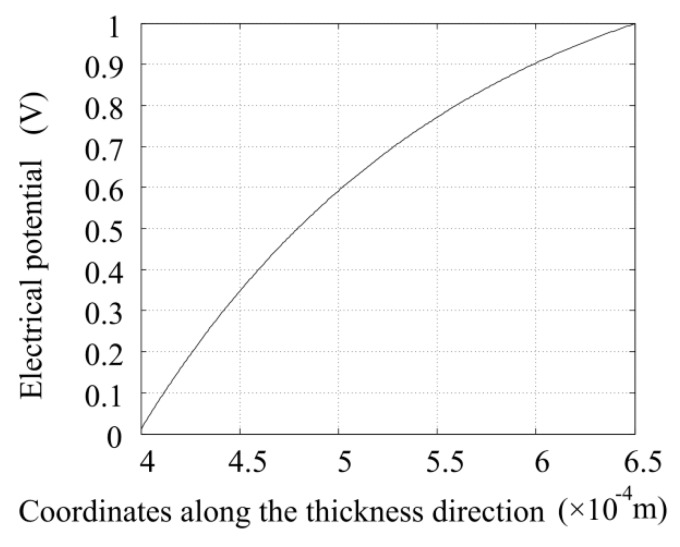
Electric potential distribution along the thickness direction when the voltage amplitude is 1 V.

Making use of Equation (10), the electric potential distribution along the radial direction is obtained. As shown in Figure 7, the electric potential is not linearly distributed along the thickness direction.

## 4. Experimental Validation

Since the displacements of the surfaces of the tubes are on nanometer scales, these deformations are structurally inside the print head. It seems impossible to measure these quantities directly. An experiment was designed to validate the above computations indirectly. When the glass tube is deformed, it will squeeze a small amount of water out of the nozzle aperture, which could be imaged by a camera with the appropriate magnification. The volume information was calculated by using image processing technology, and the volume was correlated to the deformation of the inner surface of the glass tube.

First, the print head was filled with water by using a syringe. Then, the tip part of the print head was placed into a cap that was full of water. The cap would nearly seal the front part of the print head. Afterwards, the syringe was removed from the top part of the print head and a cap was used that was prefilled with water to seal the top part of the print head firmly. Finally, the print head was installed on the experimental platform.

**Figure 8:**
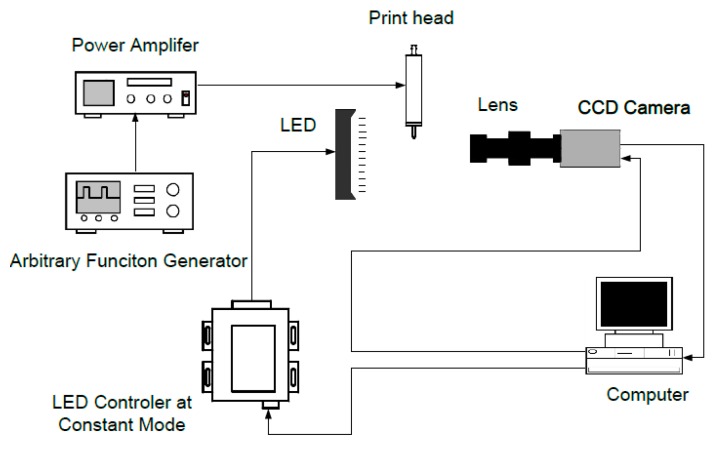
Diagram of the experimental platform.

The platform is shown in Figure 8. A step voltage signal was generated by a signal generator (Agilent 33220A, Santa Clara, CA, USA) and was amplified by a voltage amplifier. Then, it was fed to the print head. Together with a high-speed voltage output device (PCIE 6711, National Instrument, Austin, TX, USA), the computer can generate a signal with a sampling frequency of up to 1 MHz. The generated pulses were used to trigger a CCD camera (scA780-54fm/fc, Basler, Ahrensburg, Germany). A LED controller (S4000, Advanced Illumination, Rochester, MN, USA) was used to set the LED at continuous illuminating mode because the drop adhering to the nozzle is static. A lens (Moritex-ML-Z07545, Saitama, Japan) was used to properly magnify the image. The acquired image was transported to the computer for analysis through an image acquisition device (PCI-1405, National Instrument, Austin, TX, USA).

**Figure 9:**
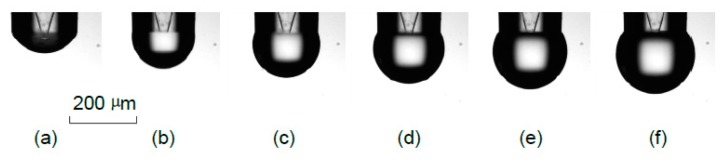
A sequence of images acquired at different voltage excitations.

Next, the bottom cap of the print head was unsealed. Through the imaging system, the print head tip was wetted with a small amount of water, as shown in Figure 9a. After the feed of voltages of 20 V, 40 V, 60 V, 80 V, 100 V, respectively, and owing to the deformation of the tubes in the print head, small amounts of water were squeezed out of the print head and imaged by the imaging system as shown in Figure 9b–f, respectively. These images were taken two seconds apart in time.

To calculate the volume of the droplet, the droplet adhered to the nozzle end face was assumed to be axisymmetric and was sliced into many layers along the vertical direction. The volume of these droplets was obtained through an image processing algorithm.

At the same time, the deformation of the glass tube was assumed to be consistent along the section that was surrounded by a piezoelectric tube, and the other segment was assumed to be without deformation. The deformation amount of the glass tube was calculated as

(30) $$V_{def}=2\pi r_{1}u_{r1}\frac{V_{inp}}{V_{ref}}l_{pie}$$

where $V_{inp}$ is the input voltage, $V_{ref}$ is the reference voltage, $u_{r1}$ is the deformation of the inner surface of the glass tube under the excitation of a reference voltage $V_{ref}$ where in this paper the reference voltage is 1.0 V (as is shown in Figure 4), and $l_{pie}$ is the length of the piezoelectric tube.

**Figure 10:**
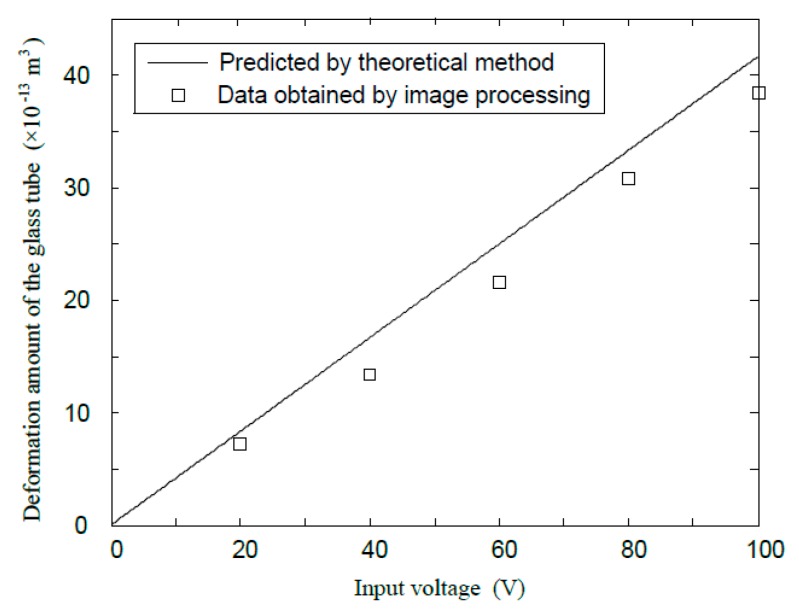
A comparison of the deformation amount by theoretical computation and image processing.

Each time a higher voltage was fed into the print head, an additional amount of water was obtained by subtracting the volume of the droplet at the higher voltage by the volume of the previous droplet. These data are compared with the result obtained by Equation (30), which is shown in Figure 10.

In Figure 10, it can be seen that the data obtained by theoretical computation and image processing agree reasonably well. The data obtained experimentally is smaller than the theoretical computation at large, which may be attributed to the bulk-modulus effect of the internal fluid and the evaporation effect of the meniscus due to its small size.

## 5. Further Discussion

**Figure 11:**
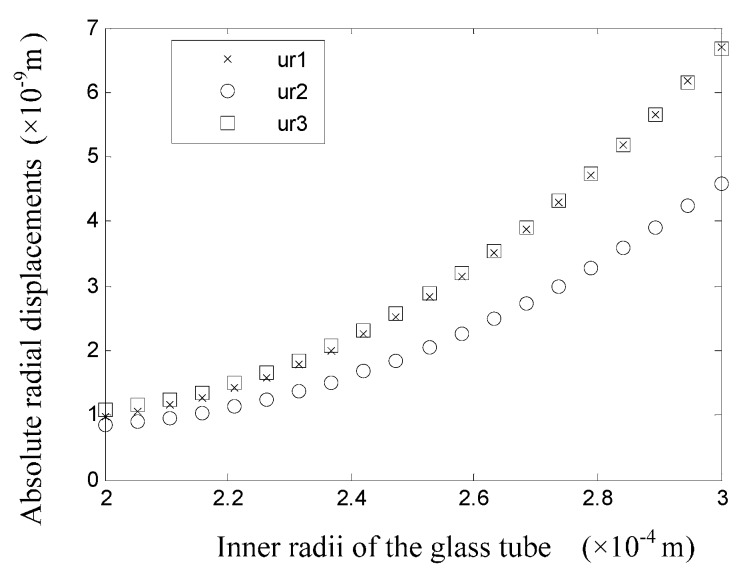
Displacements of the tube surfaces when the value of $r_{1}$ is changed.

To study the influence of tube wall thickness on the deformation and stress statuses, it is essential to investigate the displacement field when the radius of each tube is changed. As shown in Figure 11, when only the inner radius of the glass tube is changed, the wall displacements would change significantly. The symbols $u_{r1}$, $u_{r2}$, and $u_{r3}$ in Figure 11 refer to the displacements at the surfaces with a radius of $r=r_{1}$, $r=r_{2}$, and $r=r_{3}$ respectively. In addition, it is necessary to note that $u_{r1}$ refers to the displacement after the value of the inner radius of the glass tube is changed. When the value of $r_{1}$ is small, which means the thickness of the glass tube is relatively large, the displacements of the tube walls are all small. The displacements of the inner and outer surfaces of the piezoelectric tube share nearly the same displacement, and they are all larger than the displacement of the inner surface of the glass tube. When the value of $r_{1}$ increases above 0.25 mm, the glass tube becomes thinner and the system rigidity decreases. In addition, the displacement of each tube increases significantly. Moreover, the displacements of the inner surface of the glass tube and the outer surface of the piezoelectric tube are nearly the same, while the displacement of the inner surface of the piezoelectric tube is different and is the smallest.

**Figure 12:**
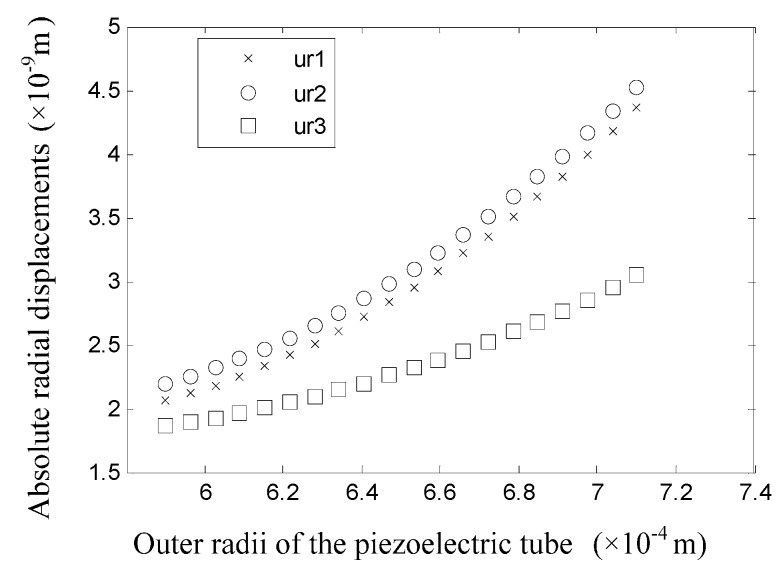
Displacements of the tube surfaces when the value of $r_{3}$ is changed.

When only the value of the outer radius of piezoelectric tube is changed, the displacements of the tube surfaces are shown in Figure 12. Note that $u_{r3}$ is the displacement of the outer surface of the piezoelectric tube after the value of $r_{3}$ is changed. It could be found that the displacements of the inner and outer surfaces of the glass tube are nearly the same. The displacement difference between the inner and outer surfaces of the piezoelectric tube increases as $r_{3}$ increases, and the inward displacement of the inner surface of the piezoelectric tube is larger than the outer surface, which means the piezoelectric tube is under a “radially elongated” state. From Figure 12, we can conclude that the drive effect is strengthened as the thickness of the piezoelectric tube is increased.

**Figure 13:**
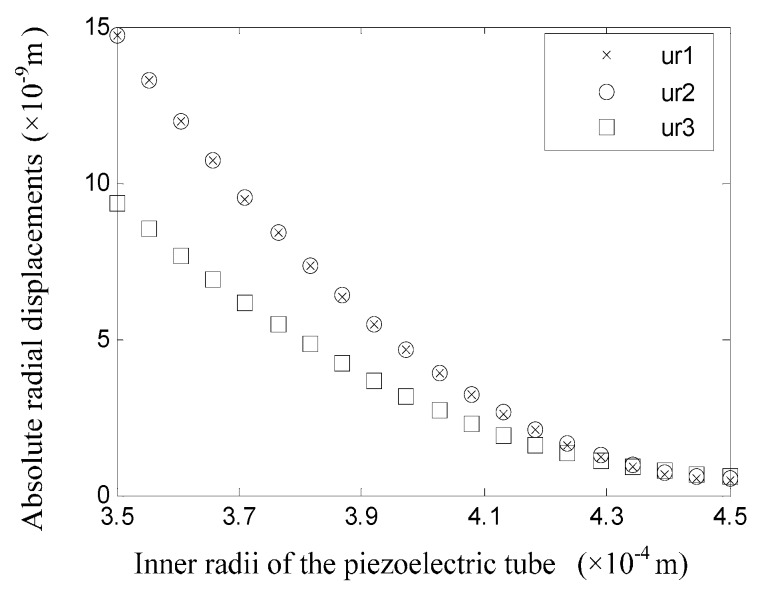
Displacements of the tube surfaces when the value of $r_{2}$ is changed.

Figure 13 shows the results of the displacements for the tube surfaces when only the value of $r_{2}$ is changed, that is, the inner radius of the piezoelectric tube and the outer radius of the glass tube are changed simultaneously. When $r_{2}$ is relatively small, in other words, when the glass tube becomes thinner and the piezoelectric tube becomes thicker, all tube walls have a larger displacement. Likely, when $r_{2}$ becomes larger, due to insufficient drive and excessive rigidity, the displacements of the tube surfaces become considerably smaller.

## 6. Conclusions

The steady state response of a tubular piezoelectric print head under the excitation of electric voltage, together with the effect of the tubes’ wall thicknesses on their deformation and stress statuses, were studied in detail in this paper. The following conclusions were obtained.

(1) When the piezoelectric tube is under the excitation of a positive step voltage, the displacements of the glass tube and the piezoelectric tube are inward towards the central axis. However, their radial stress directions are opposite. The glass tube is compressed and has an inward radial stress, while the radial stress of the piezoelectric tube is outwardly oriented.
(2) For the special tubular piezoelectric print head discussed above, the displacements of the tube walls are obtained at the several or dozens of nanometers level, which depend on the input voltage.
(3) The hoop stress is larger than the radial stress on the whole, and the potential is not linearly distributed along the radial direction.
(4) When the glass tube becomes thinner, the system has a smaller rigidity so that a larger deformation field of the tubes is obtained. Similarly, when the piezoelectric tube becomes thicker, the system’s drive effect becomes more significant so that a larger deformation field of the tubes is acquired.

## Nomenclature

$c_{11}$,$c_{13}$,$c_{33}$	elastic moduli at constant electric field;	$u_{r1}$	deformation of the inner surface of glass tube;
$D_{r}$	electric displacement vector;	$V_{def}$	deformation volume of glass tube;
$e_{31}$,$e_{33}$	piezoelectric constants;	$V_{in}$	input voltage;
E	elastic modulus;	$\varepsilon_{33}$	permittivity component at constant strain;
$l_{pie}$	length of the piezoelectric tube;	θ	circumferential coordinate;
$P_{1}$	radial stress of the inner surface piezoelectric tube;	$\gamma_{rr}$,$\gamma_{\theta \theta }$	strain;
		ΔV	amount of volume compression;
r	radial coordinate;	φ	electrical potential;
$r_{1}$,$r_{2}$,$r_{3}$	radii of glass and piezoelectric tubes;	μ	Poisson’s ratio.
$\sigma_{rr}$,$\sigma_{\theta \theta }$	stresses;	$u_{r}$	radial displacement;

## Appendix A

The property parameters of PZT-5H used in the work is shown as below.

$$\left[c\right]=\left[\begin{array}{cccccc} 12.7\times 10^{10} & 8.02\times 10^{10} & 8.47\times 10^{10} & 0 & 0 & 0\\ 8.02\times 10^{10} & 12.7\times 10^{10} & 8.47\times 10^{10} & 0 & 0 & 0\\ 8.47\times 10^{10} & 8.47\times 10^{10} & 11.7\times 10^{10} & 0 & 0 & 0\\ 0 & 0 & 0 & 2.30\times 10^{10} & 0 & 0\\ 0 & 0 & 0 & 0 & 2.30\times 10^{10} & 0\\ 0 & 0 & 0 & 0 & 0 & 2.35\times 10^{10} \end{array}\right]N/m^{2}$$

$$\left[e\right]=\left[\begin{array}{cccccc} 0 & 0 & 0 & 0 & 17.034 & 0\\ 0 & 0 & 0 & 17.034 & 0 & 0\\ -6.228 & -6.228 & 23.24 & 0 & 0 & 0 \end{array}\right]C/m^{2}$$

$$\left[\varepsilon \right]=\left[\begin{array}{ccc} 1.5\times 10^{-8} & 0 & 0\\ 0 & 1.5\times 10^{-8} & 0\\ 0 & 0 & 1.3\times 10^{-8} \end{array}\right]C^{2}/N\cdot m^{2}$$
